# Proposal and validation of a liver graft discard score for liver transplantation from deceased donors: a multicenter Italian study

**DOI:** 10.1007/s13304-022-01262-0

**Published:** 2022-03-11

**Authors:** Quirino Lai, Davide Ghinolfi, Alfonso W. Avolio, Tommaso M. Manzia, Gianluca Mennini, Fabio Melandro, Francesco Frongillo, Marco Pellicciaro, Zoe Larghi Laureiro, Rebecca Aglietti, Antonio Franco, Claudia Quaranta, Giuseppe Tisone, Salvatore Agnes, Massimo Rossi, Paolo de Simone

**Affiliations:** 1 Hepatobiliary and Organ Transplantation Unit, Department of General Surgery and Organ Transplantation, Sapienza University of Rome, Viale del Policlinico 155, 00161 Rome, Italy; 2 Division of Hepatic Surgery and Liver Transplantation, University of Pisa Medical School Hospital, Pisa, Italy; 3 Università Cattolica - Fondazione Policlinico Universitario A. Gemelli-IRCCS, Rome, Italy; 4 HPB and Transplant Unit, Department of Surgery, Tor Vergata University, Rome, Italy

**Keywords:** Donor risk index, Allocation, Graft loss, Macrovesicular steatosis, Expanded criteria donor

## Abstract

Several studies have explored the risk of graft dysfunction after liver transplantation (LT) in recent years. Conversely, risk factors for graft discard before or at procurement have poorly been investigated. The study aimed at identifying a score to predict the risk of liver-related graft discard before transplantation. Secondary aims were to test the score for prediction of biopsy-related negative features and post-LT early graft loss. A total of 4207 donors evaluated during the period January 2004–Decemeber 2018 were retrospectively analyzed. The group was split into a training set (*n* = 3,156; 75.0%) and a validation set (*n* = 1,051; 25.0%). The Donor Rejected Organ Pre-transplantation (DROP) Score was proposed: − 2.68 + (2.14 if Regional Share) + (0.03*age) + (0.04*weight)-(0.03*height) + (0.29 if diabetes) + (1.65 if anti-HCV-positive) + (0.27 if HBV core) − (0.69 if hypotension) + (0.09*creatinine) + (0.38*log10AST) + (0.34*log10ALT) + (0.06*total bilirubin). At validation, the DROP Score showed the best AUCs for the prediction of liver-related graft discard (0.82; *p* < 0.001) and macrovesicular steatosis ≥ 30% (0.71; *p* < 0.001). Patients exceeding the DROP 90th centile had the worse post-LT results (3-month graft loss: 82.8%; log-rank *P* = 0.024).The DROP score represents a valuable tool to predict the risk of liver function-related graft discard, steatosis, and early post-LT graft survival rates. Studies focused on the validation of this score in other geographical settings are required.

**Supplementary Information:**

The online version contains supplementary material available at 10.1007/s13304-022-01262-0.

## Introduction

Liver transplantation (LT) is the best therapeutic strategy for managing more than 50 pathologies causing end-stage liver disease [1]. One of the main goals of transplant physicians is to maximize the pool of available liver grafts to increase the number of transplants and reduce the number of LT candidates dying on the waiting list [2].

Therefore, the current focus is on identifying predictive criteria to guide the safe use of liver grafts [3], since inappropriate graft selection might generate fatal consequences for the recipient [4].

In recent years, many studies have focused on the risk of early graft dysfunction after transplantation [5–9], while interest has been observed in developing pre-procurement available prognosticators of scarce organ quality for transplant [3].

This study aimed at identifying and validating a score to predict the risk of liver-related graft discard from donors after brain death (DBD). The secondary aim was to test the score for prediction of biopsy-related features and graft loss at 3 months after transplantation.

## Materials and methods

### Patients

We performed a retrospective analysis of 4,372 DBDs evaluated for liver graft donation from January 1st 2004 to December. Four Italian centers joined the project: University of Pisa, Italy (*n* = 2,694), and the three University Centers of Rome (*n* = 1,678). Only DBDs offered for a primary transplant were included. DBD with missing clinical information (*n* = 17) and livers used for secondary (*n* = 118) or ABO-incompatible transplants (*n* = 30) were excluded from analysis so that the final sample numbered 4,207 cases.

This group was split into a Training Set of 3156 candidates (75.0%) and a Validation Set of 1051 candidates (25.0%) using a causal number generator randomization. A flowchart reporting the selection process is reported in Supplementary Fig. 1.

### The Italian national organ procurement and allocation system

In Italy, liver donors are allocated on a regional basis except for urgencies (i.e., fulminant hepatic failures), pediatric recipients, and patients with a model for end-stage liver disease score ≥ 29. If a local center declines a graft before or during procurement surgery, it is offered at the national level via the Italian National Center for Transplantation Office. Decline criteria are varied across centers, and liver graft biopsy is left at the discretion of the surgical procurement team. With the intent to avoid center-related biases, only donors that were declined both locally and nationally were considered in the present study.

### Definitions

We categorized the causes of graft discarding in two groups, namely liver-related versus liver-unrelated. Liver-related reasons for discard included any of the following: pre-procurement liver blood tests and/or imaging; gross anatomy; procurement histology, and poor perfusion. Liver-unrelated reasons for graft discard were donor tumors, donor infections, and pre-procurement donor cardiac arrest.

A liver graft biopsy was performed on demand, depending on surgical evaluation at procurement. The time of biopsy was before organ procurement. Biopsies review was not centralized, but performed by the different Pathology services on a rota basis.

Donor hypotension was defined as any episode of mean arterial pressure < 60 mm Hg for more than 1 h during the intensive care unit (ICU) stay.

The vasoactive-inotropic score was calculated according to the formula:

dopamine (mcg/Kg/min) + dobutamine (mcg/Kg/min) + vasopressin (U/Kg/min × 10,000) + noradrenaline (mcg/Kg/min × 100) + adrenaline (mcg/Kg/min × 100).

### Statistical analysis

Continuous variables were reported as medians and inter-quartile ranges (IQR). Dummy variables were reported as numbers and percentages. We used the maximum likelihood estimation method for managing missing data [10]. For model construction, missing data were always < 5%. Mann–Whitney *U* test and Fisher's exact test were used to compare continuous and categorical variables, respectively.

A competing-risk analysis using a cause-specific logistic regression model was constructed to identify the risk factors for liver-related graft discard. The competing event (i.e., non-liver-related graft discard) was censored in the model. The analysis was performed on the Training Set data. Thirty-one variables were initially tested in a univariable model. All the covariates with a *p* value < 0.20 were used for the multivariable model. Odds ratios (OR) and 95% confidence intervals (95%CI) were reported.

The model's accuracy was assessed through c-statistic analysis, with the intent to evaluate its ability to predict a liver-related discarded graft. In the Training Set, validation was eventually performed using a bootstrap approach based on 100 generated samples deriving from the original set.

Areas under the curve (AUCs) and 95%CIs were reported. The model's accuracy was compared in both sets with previous scores, namely the Discard Risk Index (DSRI) [3], the donor body mass index (BMI), and the donor age. The validation in the Validation Set tested sensitivity, specificity, and diagnostic odds ratio (DOR) at different thresholds of the identified score. Validation sub-analyses were done to test the score for predicting macrovescicular steatosis (MaS) > 30%, fibrosis and necrosis for donors with available liver graft histology. The Akaike information criterion (AIC) was calculated for the different scores; the lowest AIC value was associated with the best discriminatory ability for the given score [11].

Survival probabilities were estimated using the Kaplan–Meier method. Survival rates comparisons were estimated using the log-rank method. Variables with a *p* < 0.05 were considered statistically significant. We used the SPSS statistical package version 24.0 (SPSS Inc., Chicago, IL, USA).

## Results

The characteristics of the entire population, Training and Validation Sets are reported in Table 1. Overall, 2,642/4,207 (62.8%) grafts were considered eligible for LT and 1565 (37.2%) were discarded. Liver-related issues were the reason for graft discard in 1254 cases (29.8%) versus liver-unrelated in 311 (7.4%). In the liver-related group, the most common reasons for declining a graft were: poor histology (*n* = 660; 15.7%); pre-procurement liver function tests and/or imaging (*n* = 310; 7.4%); poor macroscopic aspect of the organ at surgery (*n* = 216; 5.1%); and poor perfusion during procurement (*n* = 68; 1.6%).

**Table 1:** Characteristics of the donors enrolled in the study

Variables	Entire population (*N* = 4,207; 100.0)	Training set (*n* = 3,156; 75.0)	Validation set (*n* = 1,051; 25.0)	*p*
	Median (IQR) or *n* (%)			
Age (years)	66 (51–76)	66 (51–76)	66 (50–76)	0.90
Male gender	2,298 (54.6)	1,721 (54.5)	577 (54.9)	0.86
Caucasian ethnicity	4,147 (98.6)	3,113 (98.6)	1,034 (98.4)	0.55
Weight (kg)	75 (65–82)	75 (65–82)	75 (65–84)	0.50
Height (cm)	170 (162–175)	170 (162–175)	170 (162–175)	0.89
BMI	26 (24–28)	26 (23–28)	26 (24–28)	0.49
Anti-HCV positive	140 (3.3)	107 (3.4)	33 (3.1)	0.77
HBV core positive	694 (16.5)	507 (16.1)	187 (17.8)	0.20
Cause of death				
Trauma	889 (21.1)	663 (21.0)	226 (21.5)	0.73
Anoxia	206 (4.9)	154 (4.9)	52 (4.9)	0.93
CVA	2,991 (71.1)	2,251 (71.3)	740 (70.4)	0.58
Other	113 (2.7)	81 (2.6)	32 (3.0)	0.44
History of morbidity				
DM2	520 (12.4)	404 (12.8)	116 (11.0)	0.14
Hypertension	2,083 (49.5)	1,574 (49.9)	509 (48.4)	0.43
Cardiopathy	1,154 (27.4)	882 (27.9)	272 (25.9)	0.20
Dyslipidemia	596 (14.2)	441 (14.0)	155 (14.7)	0.54
ICU length of stay (days)	3 (2–5)	3 (2–5)	3 (2–5)	0.46
Regional sharing	2,036 (48.4)	1,529 (48.4)	507 (48.2)	0.92
Hypotensive episode(s)	1,387 (33.0)	1,032 (32.7)	355 (33.8)	0.52
Cardiac arrest(s)	391 (9.3)	276 (8.7)	115 (10.9)	0.04
Inotropes use	3,383 (80.4)	2,519 (79.8)	864 (82.2)	0.10
Noradrenaline	2,594 (61.7)	1,937 (61.4)	657 (62.5)	0.53
Adrenaline	150 (3.6)	102 (3.2)	48 (4.6)	0.054
Vasopressine	73 (1.7)	53 (1.7)	20 (1.9)	0.68
Dopamine	1,246 (29.6)	922 (29.2)	324 (30.8)	0.33
Dobutamine	176 (4.3)	131 (4.2)	45 (4.3)	0.86
More than one drug used	806 (19.2)	586 (18.6)	220 (20.9)	0.09
VAS	10 (3–30)	10 (3–30)	12 (4–30)	0.19
Serum creatinine peak (mg/dL)	1.1 (0.8–1.5)	1.1 (0.8–1.5)	1.1 (0.8–1.5)	0.43
Sodium peak (mEq/L)	151 (145–157)	151 (145–157)	151 (145–157)	0.31
AST peak (IU/L)	38 (24–71)	38 (24–71)	38 (24–68)	0.82
ALT peak (IU/L)	29 (18–58)	29 (18–58)	28 (18–56)	0.75
Total bilirubin peak (mg/dL)	0.8 (0.5–1.1)	0.7 (0.5–1.1)	0.8 (0.5–1.1)	0.52
DSRI	3.3 (2.5–4.7)	3.3 (2.5–4.7)	3.3 (2.4–4.7)	0.61
Biopsy	1,975 (46.9)	1,479 (46.9)	496 (47.2)	0.86
Graft discarded	1,565 (37.2)	1,169 (37.0)	396 (37.7)	0.71
Liver-related	1,254 (29.8)	936 (29.7)	318 (30.3)	0.73
on chart	310 (7.4)	223 (7.1)	87 (8.3)	0.20
macroscopic aspect	216 (5.1)	161 (5.1)	55 (5.2)	0.87
poor perfusion	68 (1.6)	47 (1.5)	21 (2.0)	0.26
biopsy	660 (15.7)	505 (16.0)	155 (14.7)	0.35
No liver-related	311 (7.4)	233 (7.4)	78 (7.4)	0.95
group match	10 (0.2)	8 (0.3)	2 (0.2)	1.00
size match	10 (0.2)	5 (0.2)	5 (0.5)	0.13
organizational	97 (2.3)	74 (2.3)	23 (2.2)	0.91
tumor	111 (2.6)	87 (2.8)	24 (2.3)	0.44
cardiac arrest	17 (0.4)	12 (0.4)	5 (0.5)	0.78
bacterial infection	66 (1.6)	47 (1.5)	19 (1.8)	0.48
Graft transplanted	2,642 (62.8)	1,987 (63.0)	655 (62.3)	0.71

*IQR* inter-quartile ranges, *BMI* body mass index, *HCV* hepatitis C virus, *HBV* hepatitis B virus, *CVdA* cerebro-vascular accident, *DM2* diabetes mellitus type-II, *ICU* intensive care unit, *VAS* vasoactive score, *AST* aspartate transaminase, *ALT* alanine transaminase, *DSRI* Discard Risk Index

Among the liver-unrelated discard causes the most frequent were: tumors (*n* = 111; 2.6%); bacterial infections (*n* = 66; 1.6%); pre-procurement cardiac arrest (*n* = 17; 0.4%).

**Fig. 1:**
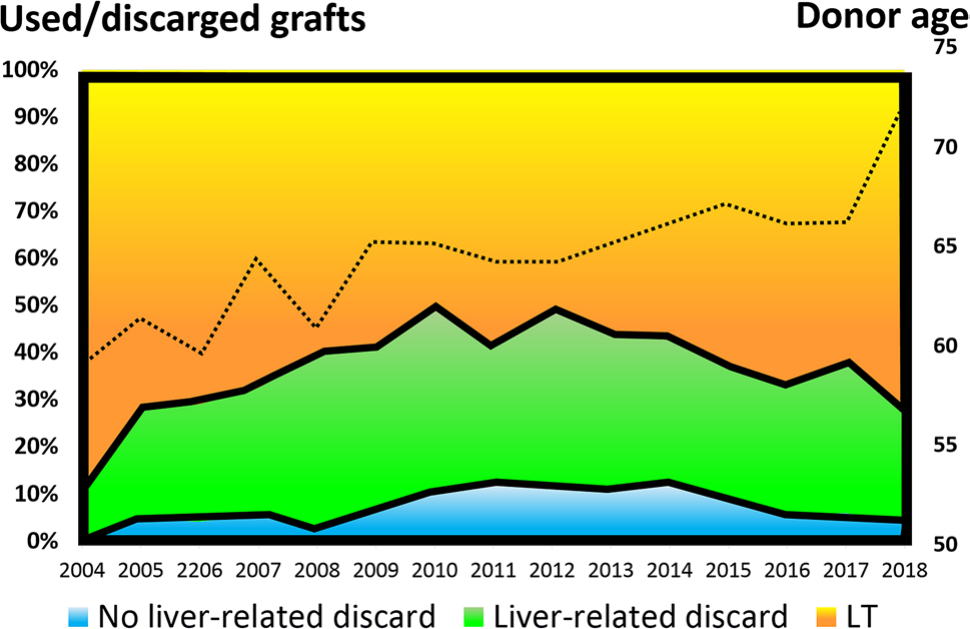
Diagram showing the different rates of discarded/used grafts observed during the entire study period, and the progressive increase of the median donor age during the same period

The rates of discarded and used grafts throughout the study period are reported in Fig. 1, and the rates of liver-related versus liver-unrelated causes of discard and the median donor age.

As reported in Table 1, the median donor age of the entire donor population was 66 years (IQR: 51–76). Anti-hepatitis B virus (HBV) core antigen and anti-HCV-positive donors were reported in 694 (16.5%) and 140 (3.3%) cases. Donors death was due to cerebro-vascular accident in 2991 cases (71.1%); trauma in 889 (21.1%); anoxia in 206 (4.9%), and to other causes in 113 (2.7%). The most frequent donor co-morbidities were arterial hypertension in 2083 cases (49.5%); cardiac disease of any origin in 1154 (27.4%); dyslipidemia in 596 (14.2%), and diabetes mellitus type-II (DM2) in 520 (12.4%). AST and ALT median peak values were 38 (IQR: 24–71) and 29 IU/L (IQR: 18–58), respectively. The median total bilirubin peak was 0.8 mg/dL (IQR: 0.5–1.1). Liver graft histology was obtained in 1975 (46.9%) donors.

### Risk factors for liver-related graft discard

Table 2 illustrates the results of the uni- and multivariable logistic regression analyses for risk factors of liver-related graft discard. At multivariable analysis, these were regional share (OR = 8.46; *p* < 0.001); anti-HCV-positive donor (OR = 5.19; *p* < 0.001); weight (OR = 1.04; *p* < 0.001), and age (OR = 1.03; *p* < 0.001). Anti-HBV core positive donor (OR = 1.31; *p* = 0.03) and DM2 (OR = 1.33; *p* = 0.04) were also risk factors. On the opposite, previous hypotension episodes (OR = 0.50; *p* < 0.001) and donor height (OR = 0.97; *p* < 0.001) showed a protective effect for the risk of liver-related graft discard.

**Table 2:** Uni- and multivariable logistic regression analyses for the risk of liver-related graft discard

Variables	Univariable analysis					Multivariable analysis				
	Beta	SE	OR	95%CI	*p*	Beta	SE	OR	95%CI	*p*
Regional share	2.13	0.10	8.45	7.01–10.19	<0.001	2.14	0.10	8.46	6.92–10.34	<0.001
Age	0.02	0.00	1.02	1.02–1.03	<0.001	0.03	0.00	1.03	1.02–1.03	<0.001
Male gender	− 0.03	0.08	0.97	0.84–1.14	0.73	–	–	–	–	–
Caucasian	− 0.24	0.32	0.78	0.42–1.48	0.45	–	–	–	–	–
Trauma as cause of death	− 0.43	0.10	0.65	0.53–0.79	<0.001	–	–	–	–	–
Anoxia as cause of death	0.35	0.17	1.42	1.01–1.99	0.04	–	–	–	–	–
CVA as cause of death	0.22	0.09	1.25	1.05–1.49	0.01	–	–	–	–	–
ICU length of stay	− 0.00	0.01	1.00	0.98–1.01	0.68	–	–	–	–	–
Weight	0.03	0.00	1.03	1.02–1.03	<0.001	0.04	0.00	1.04	1.03–1.05	<0.001
Height	− 0.01	0.00	0.99	0.99–1.00	0.18	− 0.03	0.01	0.97	0.96–0.98	<0.001
BMI	0.11	0.01	1.11	1.09–1.13	<0.001	–	–	–	–	–
DM2	0.67	0.11	1.95	1.58–2.42	<0.001	0.29	0.14	1.33	1.02–1.74	0.04
Hypertension	0.58	0.08	1.79	1.54–2.10	<0.001	–	–	–	–	–
Cardiopathy	0.43	0.08	1.53	1.30–1.81	<0.001	–	–	–	–	–
Dyslipidemia	0.25	0.11	1.28	1.03–1.59	0.02	–	–	–	–	–
HCV-positive	1.83	0.22	6.24	4.08–9.55	<0.001	1.65	0.25	5.19	3.18–8.47	<0.001
HBV core	0.43	0.10	1.53	1.25–1.87	<0.001	0.27	0.12	1.31	1.04–1.66	0.03
Hypotension episode(s)	− 0.88	0.09	0.42	0.35–0.50	<0.001	− 0.69	0.11	0.50	0.40–0.62	<0.001
Cardiac arrest(s)	0.19	0.13	1.21	0.93–1.57	0.16	–	–	–	–	–
Noradrenaline use	− 0.02	0.08	0.98	0.84–1.15	0.84	–	–	–	–	–
Adrenaline use	0.22	0.21	1.25	0.82–1.89	0.30	–	–	–	–	–
Vasopressine use	− 0.88	0.39	0.42	0.20–0.89	0.02	–	–	–	–	–
Dopamine use	0.04	0.09	1.04	0.88–1.23	0.63	–	–	–	–	–
Dobutamine use	0.04	0.19	1.04	0.71–1.53	0.82	–	–	–	–	–
VAS	0.00	0.00	1.00	1.00–1.00	0.28	–	–	–	–	–
More than one drug used	0.05	0.10	1.05	0.87–1.28	0.60	–	–	–	–	–
Serum creatinine peak	0.14	0.04	1.15	1.07–1.25	<0.001	0.09	0.05	1.10	1.00–1.21	0.055
Sodium peak	− 0.01	0.00	0.99	0.98–1.00	0.02	–	–	–	–	–
Log_10_AST peak	0.31	0.10	1.37	1.13–1.66	0.001	0.38	0.20	1.46	0.99–2.15	0.057
Log_10_ALT peak	0.26	0.09	1.29	1.08–1.55	0.006	0.34	0.19	1.40	0.97–2.03	0.07
Total bilirubin peak	0.05	0.03	1.05	0.99–1.11	0.08	0.06	0.03	1.06	1.00–1.13	0.059
Constant	–	–	–	–	–	− 2.68	1.03	0.07	–	0.01

− 2Loglikelihood: 2,876.7

*SE* standard error, *OR* odds ratio, *CI* confidence intervals, *CVA* cerebro-vascular accident, *ICU* intensive care unit, *BMI* body mass index, *DM2* diabetes mellitus type-II, *HCV* hepatitis C virus, *HBV* hepatitis B virus, *VAS* vasoactive score, *AST* aspartate transaminase, *ALT* alanine transaminase

Peak values of serum creatinine (OR = 1.10; *p* = 0.055), AST (OR = 1.46; *p* = 0.057), total bilirubin (OR = 1.06; *p* = 0.059), and ALT (OR = 1.40; *p* = 0.07) were slightly below the level of statistical significance.

Using the beta-coefficients derived from the multivariable model, we constructed the following score:

Donor Rejected Organ Pre-transplantation (DROP) Score = − 2.68 + (2.14 if Regional Share) + (0.03*age years) + (0.04*weight kg)—(0.03*height cm) + (0.29 if DM2) + (1.65 if anti-HCV positive) + (0.27 if anti-HBV core positive)—(0.69 if hypotension episode) + (0.09*serum creatinine peak) + (0.38*log10 AST peak) + (0.34*log10 ALT peak) + (0.06*total bilirubin peak).

### Validation for the risk of liver-related graft discard

The DROP Score was tested in both the Training and Validation Sets for prediction of the risk of liver-related graft discard. DROP showed a higher AUC (0.83 and 0.82; *p* < 0.001) concerning the other tested scores in both validation processes. For instance, DSRI AUC was 0.66–0.68, while donor BMI and donor age AUCs were 0.62 and 0.59–0.61, respectively (Table 3). Again, in terms of AIC DROP showed better accuracy and smaller values. In the Training Set validation process, DROP AIC was 2,877.87 versus 3,727.60 for DSRI. In the Validation Set validation process, its AIC was 976.62 vs. 1,253.56.

**Table 3:** Validation of the score: ROC analysis for the prediction of liver-related graft discard

Scores	Training set				Validation set			
	AUC	SE	95%CI	AIC	AUC	SE	95%CI	AIC
DROP	0.83	0.01	0.81–0.84	2,877.87	0.82	0.01	0.79–0.85	976.62
DSRI	0.66	0.01	0.64–0.68	3,727.60	0.68	0.02	0.65–0.72	1,253.56
Donor BMI	0.62	0.01	0.59–0.64	3,715.63	0.62	0.02	0.58–0.65	1,249.82
Donor age	0.59	0.01	0.56–0.61	3,757.65	0.61	0.02	0.57–0.64	1,251.36

DROP centiles	Liver-related discarded grafts %		Score value	Sensitivity	Specificity	DOR
10	0–10	3.4	− 2.55	98.7	13.6	0.16
20	11–20	5.0	− 2.05	96.9	27.3	0.38
25	21–25	7.7	− 1.86	95.9	34.0	0.52
30	26–30	13.8	− 1.66	92.1	39.6	0.66
40	31–40	12.8	− 1.28	88.1	52.1	1.088
50	41–50	15.7	− 0.79	82.4	64.0	1.78
60	51–60	22.4	− 0.08	75.8	75.4	3.07
70	61–70	39.2	0.42	61.9	84.0	5.25
75	71–75	48.1	0.66	54.7	88.0	7.33
80	76–80	59.8	0.83	43.7	90.3	9.31
90	81–90	59.8	1.32	26.4	97.1	33.48
	91–100	76.8				

*AUC* area under the curve, *SE* standard error, *CI* confidence intervals, *AIC* Akaike information criterion, *DROP* Donor Rejected Organ Pre-transplantation, *DSRI* Discard Risk Index, *BMI* body mass index, *DOR* diagnostic odds ratio

After stratification of DROP scores in deciles, different thresholds were investigated. A value corresponding to the 50th centile was identified as a low DROP value. A value corresponding to the 90th centile (hence, high DROP) showed the best DOR (33.48) with a sensitivity of 26.4 and a specificity of 97.1 (Table 3). Supplementary Fig. 2 illustrates the percent of DBDs with low versus high DROP scores throughout the study period.

### Validation for the risk of MaS, fibrosis, and necrosis

The DROP was tested in both the Training and Validation Sets to predict macrovesicular steatosis (MaS) > 30% and any rate of fibrosis and necrosis. In both the sets, DROP AUC and AIC performed better than the tested scores (Table 4). Supplementary Fig. 3 shows a direct correlation between higher DROP scores and the severity of histology-proven graft lesions.

**Table 4:** Validation of the score: ROC analysis for the prediction of MaS ≥ 30%, any rate of fibrosis, and necrosis (analysis performed only for grafts with histology)

Scores	Training set				Validation set			
	AUC	SE	95%CI	AIC	AUC	SE	95%CI	AIC
MaS ≥ 30%								
DROP	0.68	0.02	0.65–0.71	1,579.84	0.71	0.03	0.66–0.77	482.15
Donor BMI	0.64	0.02	0.61–0.67	1,636.45	0.64	0.03	0.58–0.70	513.41
DSRI	0.53	0.02	0.49–0.56	1,695.93	0.54	0.03	0.48–0.60	532.30
Donor age	0.40	0.02	0.37–0.43	1,680.79	0.44	0.03	0.38–0.50	533.62
Fibrosis (any rate)								
DROP	0.66	0.02	0.63–0.69	1,607.92	0.66	0.03	0.60–0.71	563.84
DSRI	0.56	0.02	0.53–0.60	1,686.65	0.57	0.03	0.51–0.62	590.76
Donor age	0.53	0.02	0.50–0.56	1,687.81	0.55	0.03	0.49–0.60	595.53
HCV	0.52	0.03	0.46–0.57	1,661.37	0.52	0.03	0.47–0.58	586.62
HBV core	0.51	0.03	0.45–0.56	1,694.14	0.51	0.03	0.46–0.57	595.48
Necrosis (any rate)								
DROP	0.67	0.03	0.62–0.72	814.99	0.65	0.04	0.58–0.73	301.21
AST	0.61	0.03	0.55–0.67	838.40	0.64	0.05	0.55–0.72	309.50
ALT	0.63	0.03	0.57–0.69	829.55	0.63	0.05	0.54–0.73	306.39
Donor age	0.51	0.03	0.46–0.56	853.53	0.40	0.04	0.31–0.48	312.13
DSRI	0.63	0.03	0.58–0.68	837.82	0.56	0.05	0.47–0.65	312.45

*AUC* area under the curve, *SE* standard error, *CI* confidence intervals, *AIC* Akaike information criterion, *MaS* macrovesicular steatosis, *DROP* Donor Rejected Organ Pre-transplantation, *BMI* body mass index, *DSRI* Discard Risk Index, *HCV* hepatitis C virus, *HBV* hepatitis B virus, *AST* aspartate transaminase, *ALT* alanine transaminase

### Post-transplant graft loss evaluation

**Fig. 2:**
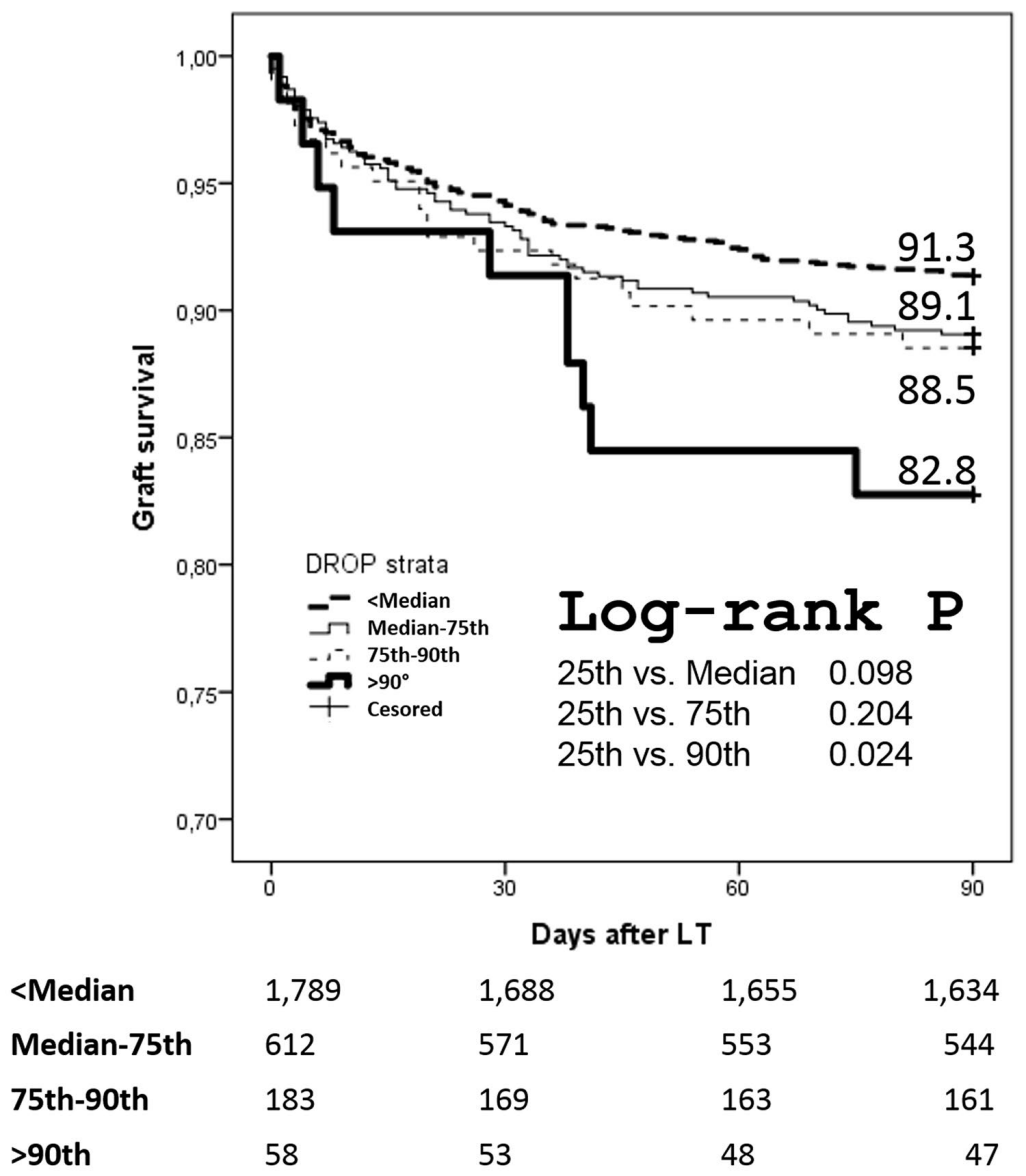
Three-month post-transplant graft survival rates according to DROP score stratification

The group of recipients of the Training and Validation Sets (*n* = 2,642) were stratified in four sub-classes according to the following thresholds of DROP: <50th centile (<− 0.79); 50th–75th centile (− 0.79 to 0.66); 75th–90th centile (0.66–1.32); and, >90th centile (>1.32). At survival analyses, patients >the 90th centile of DROP showed 3-month worse survival versus the <50th centile (82.8 vs. 91.3%; log-rank *p* = 0.024) (Fig. 2). In Supplementary Fig. 4, a sub-analysis has been reported in which the transplanted patients were dichotomized in two different time period: 2004–2010 and 2011–2018. Interestingly, the differences among the different DROP classes disappeared in the more recent period.

## Discussion

The present study illustrates a new score for the prediction of the risk of DBD liver-related graft discard. To the best of our knowledge, DROP is the first score developed with this specific aim. Several scores reported previously in the international literature have focused more on donor-specific features [3], or on a combination of donor- and transplant-related variables [5–9, 12, 13]. Among them, the Donor Risk Index (DRI) has internationally been recognized as a valuable tool for liver graft selection [12]. However, broad implementation of DRI is limited by its being sensitive to the geographical setting where it was derived [14], and by including variables (i.e., cold ischemia time ([CIT]) that can be obtained only at transplantation [15]. A recent European-derived score—namely the Euro-Transplant-DRI (ET-DRI)—recalibrated DRI score coefficients according to the European epidemiology, but this score also includes CIT and can be obtained only after transplantation. [13].

A score focusing on the risk of liver graft discarding—namely, the discard risk index (DSRI)—has recently been generated from a large donor population (*n* = 72,297) and based entirely on pre-transplant variables [3]. However, DSRI includes procured donors only (i.e., those undergoing procurement surgery) and excludes discarded donors as per pre-procurement imaging and/or blood test results [3]. A further limitation to DSRI might be that no distinction between liver-related and unrelated causes of discard has been made [3].

We developed the DROP score able to solve the above limitations. Created on an extensive, interregional experience (*n* = 3,156 DBDs) and validated on a Training Set of 1,051 donors, DROP was entirely based on variables available at the time of donor reporting and included not only donors discarded after intra-operative evaluation but also those declined before procurement as per their clinical chart data. Since graft discard criteria may greatly vary across transplant centers due to local experience and waitlist dynamics, the current study included only liver grafts that were declined at a national level. However, this might not have entirely offset the bias of initial graft discard on the eventual decline by other centers, as highlighted in the international literature [16, 17]. Moreover, the study spans over 15 years (2004–2018), and specific time-dependent biases (i.e., increasing experience with extended criteria donors or the introduction of ex situ machine perfusion) might have changed transplant centers policies [18].

Furthermore, DROP primary aim was the prediction of liver function-related discard. Thus, we reduced the impact of biases related to donors dropped off for other causes like tumors, bacterial infections, or organizational issues.

The DROP score identified 12 different variables for its construction. In some cases, the reason why the selected variables were significant was conceptually logical. For example, donor age, donor weight, and history of DM2 might portend more severe MaS. In agreement with this finding, previous reports have confirmed their role in predicting a higher risk of graft loss [19], and biliary complications [20]. Donor height might be another surrogate of graft quality and play an inverse role than donor weight: in other words, the higher the donor, the lower the risk of graft discard. This observation is consistent with the results observed in the DRI and ET-DRI studies. [12, 13].

Some variables included in the model are historical. Until the introduction of direct-acting antivirals, donor HCV-positive status was a strong surrogate of underlying liver disease, fibrosis, and inflammation [21]. Several studies have reported the use of HCV-positive grafts, mainly from RNA-negative donors [22, 23]. The weight of this variable will likely disappear in the next years. Consistently, anti-HBV core positivity might be a surrogate of poor graft quality. Previous studies highlighted a negative impact of donor anti-HBc positivity on post-transplant survival [24, 25]. Again, the role of anti-HBc core positivity is anticipated to decline in the following years, requiring recalibration of the score.

Higher peak values of AST, ALT, and total bilirubin might portend more severe ischemia–reperfusion injury, graft necrosis, or be the result of donor hemodynamic instability. Accordingly, serum creatinine is sensitive to hemodynamics, fluid, and electrolyte balance and might be strictly correlated with liver graft quality. The role of all these variables on transplant outcome has already been substantiated to a considerable extent. [3, 12, 13].

The role of other variables included in the DROP is less clear. As an example, regional sharing turned out to be a risk factor versus the extra-regional one. This finding seems somewhat contradictory to other scores like DRI, where the greater the distance, the higher the risk of poor organ quality [12]. However, DROP and DRI have been developed with different aims, and DRI focuses on the risk of poor post-transplant survival [12]. Consequently, donors procured far from the transplant center have longer CIT and weaker results [26]. On the opposite, DROP was developed to investigate the risk of liver-related graft discard. The negative role of regional sharing on liver graft decline might be due to similar evaluation criteria across regional centers, while extra-regional donors are usually accepted for priority patients (i.e., national urgencies). In other words, centers are more willing to accept all regional local donors on the chart and decline the livers according to biopsy or gross appearance. At the same time, a more accurate selection takes place during the call offer of an extra-regional donor, with the intent to avoid unnecessary travels, higher costs, and loss of human resources.

A paradoxical result of the score is the protective role of donor hypotension episodes. Three possible explanations might account for this result. First, donors with previous hypotension episodes might require more accurate hemodynamic control during the agonic phase with resulting improved organ perfusion [27]. Second, donors with prolonged hypotension episodes typically show generalized organ failure and are excluded from the donation, while donors with hypotension episodes that are still considered for donation are intensively managed. Finally, a preconditioning role of hypotension cannot be excluded in these donors, thus minimizing the impact of ischemia–reperfusion injury. [28].

A relevant aspect of the score was its ability to predict the results of the graft histology. For example, the AUC for the diagnosis of MaS ≥ 30% was 0.71 in the Validation Set. In other terms, the score identified seven out of ten donors with MaS < 30% (true negative) or ≥ 30% (true positive). Also the AIC was the best one among the different tested scores. AIC estimates the relative amount of information lost by a given model: the less information a model loses, the higher the quality. In other terms, the smallest the AIC value, the smallest the loss of information, the better the quality of the model. It is fascinating to note that a mathematical score that can be obtained with data available at the time of donor reporting can predict the risk of MaS with similar diagnostic performances of gross evaluation of expert procurement surgeons or radiological examinations performable only during procurement [29, 30].

The practical use of this score should present several beneficial effects, mainly in the setting of a more appropriate donor–recipient matching. In Italy, in fact, the transplant centers are not strictly bound to a MELD-based allocation system, presenting a percentage of cases in which a proper allocation of marginal offers to fitter recipients is done and vice versa [31]. The effect of this improved allocation process should be already supposed observing the results of Supplementary Fig. 4, in which acceptable 3-month results were observed in recent years also when grafts with high DROP value were transplanted.

The further amelioration of the donor–recipient match vesiculated by the early identification during the donation process of grafts with a relevant risk of discard should better consent to allocate them only to specific sub-groups of recipients presenting a beneficial effect in receiving even more marginal grafts (i.e., advanced HCC, colorectal metastases).

The study presents some limitations. First, it is based on retrospective and multicenter data. Nevertheless, these biases are shared by all the studies focusing on this topic [3, 12, 13]. Unfortunately, the retrospective nature of the study limited our ability to collect all the required information about important issues like the results of the pre-donation imaging. We are confident that future studies aimed at recalibrating the score should be done adding these parameters. Second, the decision to accept an organ is often dependent on specific prerogatives of the center. With the intent to overcome this limit, we tried to minimize center-specific biases, including only organs discarded on a national basis.

Third, the liver biopsies performed before organ procurement were not evaluated by the same pathologists, and an interrater variability assessment for macrovesicular steatosis should be considered. Unfortunately, the possibility to perform a centralized revision of the biopsies was impossible due to the retrospective nature of the study.

Lastly, we are not able to assert if the two Italian regions considered in the present study had a different rate of graft discard respect to the national mean value. This datum should impact mainly on the role of the variable “regional sharing” as a risk factor for liver-related graft discard. Further studies involving more centers are required for better detailing this aspect.

In conclusion, the DROP score might be a useful tool to predict the risk of liver-related graft discard. The score is also able to predict several histological variables like steatosis, fibrosis, and necrosis. More studies aimed at investigating this score in other geographical settings are required.

## Supplementary Information

Below is the link to the electronic supplementary material.

Supplementary file1 (DOC 29 KB)

Supplementary file2 (TIF 329 KB)

Supplementary file3 (TIF 317 KB)

Supplementary file4 (TIF 423 KB)

Supplementary file5 (TIF 7323 KB)

## Abbreviations

AIC: Akaike information criterion
AUC: Area under the curve
BMI: Body mass index
CI: Confidence intervals
CIT: Cold ischemia time
DBD: Donor after brain death
DM2: Diabetes mellitus type-II
DOR: Diagnostic odds ratio
DRI: Donor Risk Index
DROP: Donor rejected organ pre-transplantation
DSRI: Discard Risk Index
ET-DRI: Euro-transplant-DRI
HBV: Hepatitis B virus
HCV: Hepatitis C virus
IQR: Interquartile ranges
LT: Liver transplantation
MaS: Macrovesicular steatosis
OR: Odds ratio
