# Supplementary material for: Proposal and validation of a liver graft discard score for liver transplantation from deceased donors: a multicenter Italian study
Source: Updates Surg. 2022 Mar 11;74(2):491–500. doi: 10.1007/s13304-022-01262-0 (PMC8995238; doi:10.1007/s13304-022-01262-0)
Supplement: Supplementary file 1 — Supplementary file1 (DOC 29 KB) [file 13304_2022_1262_MOESM1_ESM.doc]

**SUPPLEMENTARY FIGURES**

**Supplementary** **Figure 1**. Flowchart reporting the selection process performed in the study.

**Supplementary** **Figure 2.** Time-dependent modification of donors with low versus high DROP scores.

**Supplementary Figure 3.** Correlation between the higher DROP score values and the presence of advanced MaS, fibrosis or necrosis.

**Supplementary Figure 4.** A) three-month post-transplant graft survival rates according to DROP score stratification in the first period (2004-2010); B) three-month post-transplant graft survival rates according to DROP score stratification in the second period (2011-2018).
