# Supplementary figures and images for: Proposal and validation of a liver graft discard score for liver transplantation from deceased donors: a multicenter Italian study
Source: Updates Surg. 2022 Mar 11;74(2):491–500. doi: 10.1007/s13304-022-01262-0 (PMC8995238; doi:10.1007/s13304-022-01262-0)

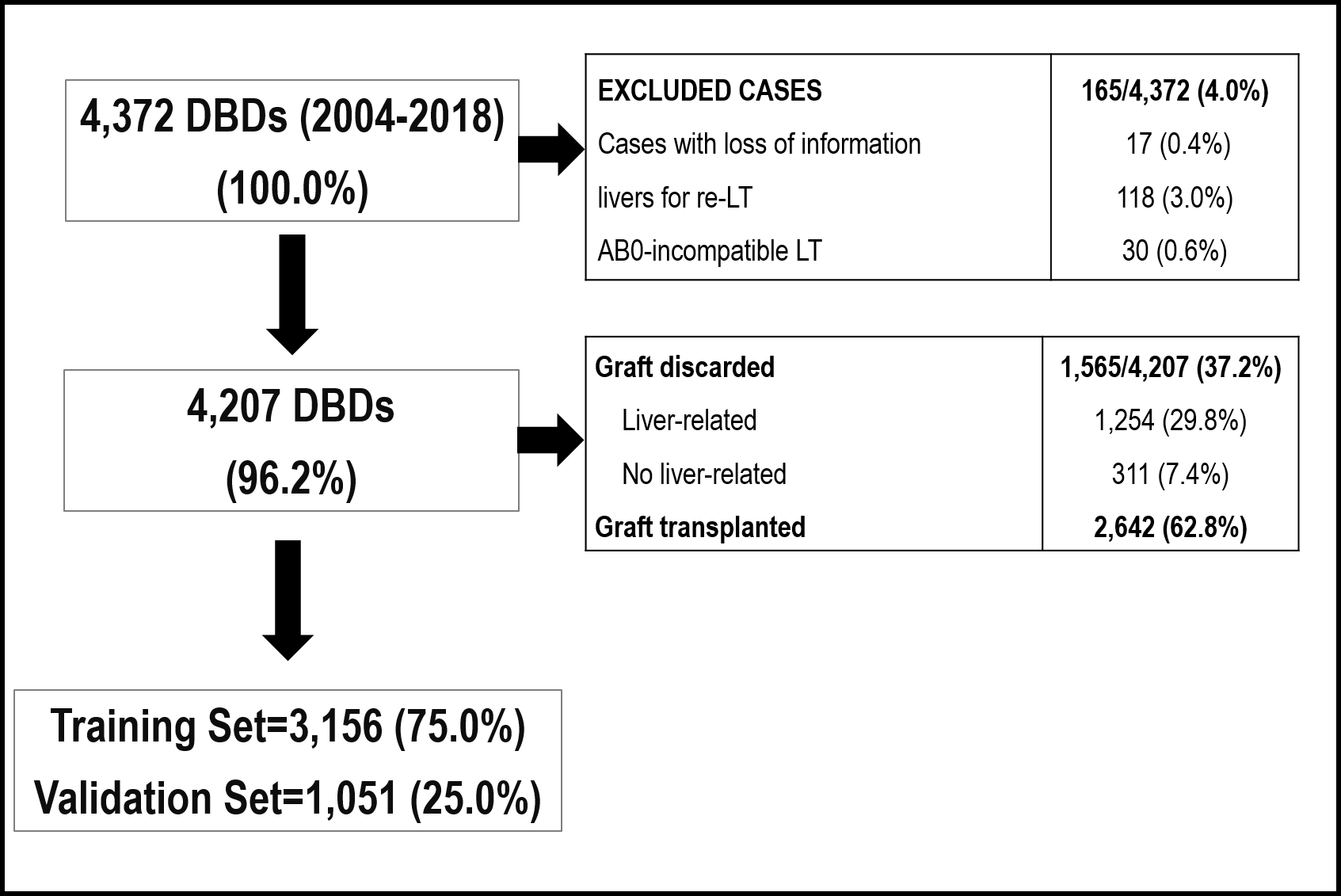

Supplement: Supplementary file 2 — Supplementary file2 (TIF 329 KB) [file 13304_2022_1262_MOESM2_ESM.tif]

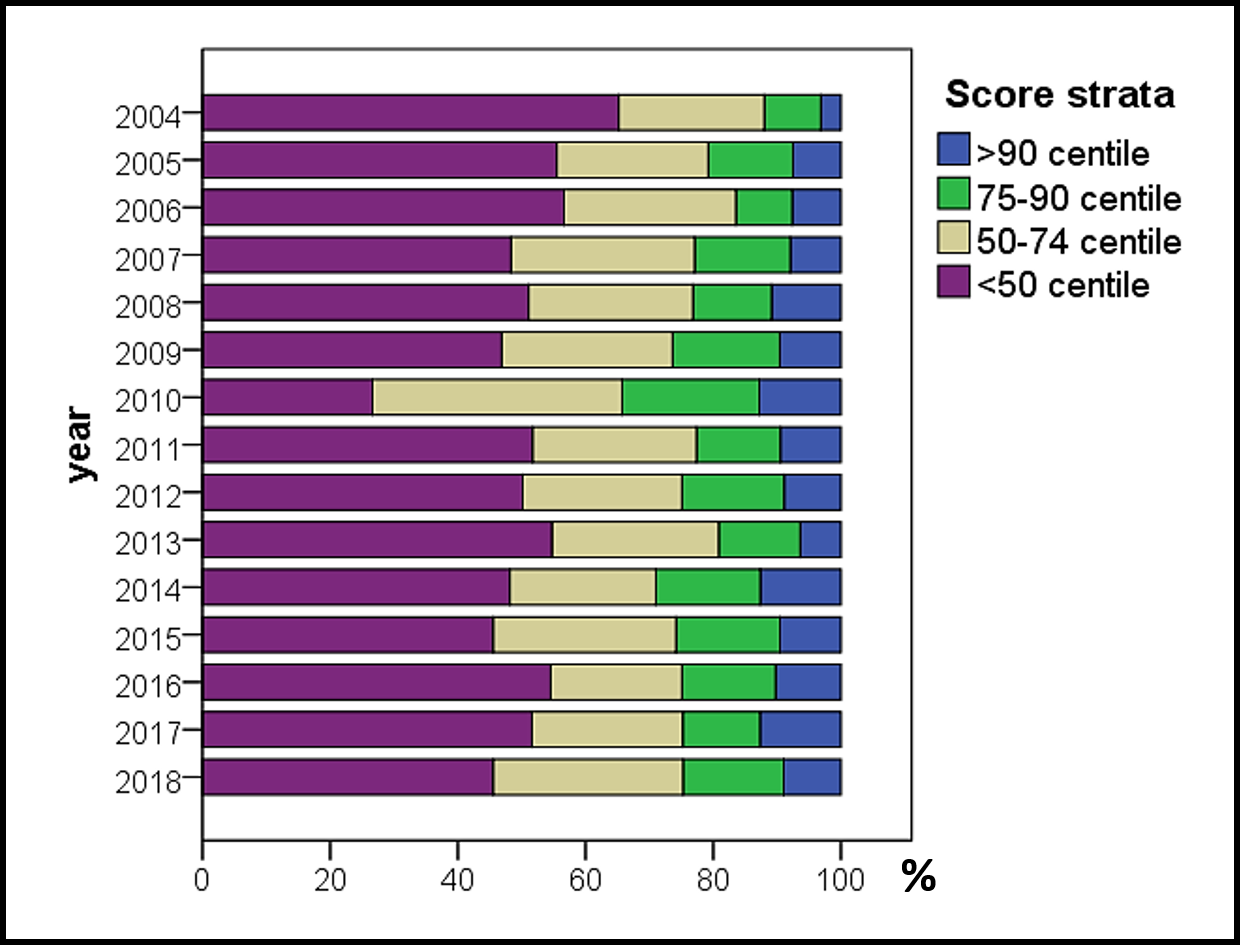

Supplement: Supplementary file 3 — Supplementary file3 (TIF 317 KB) [file 13304_2022_1262_MOESM3_ESM.tif]

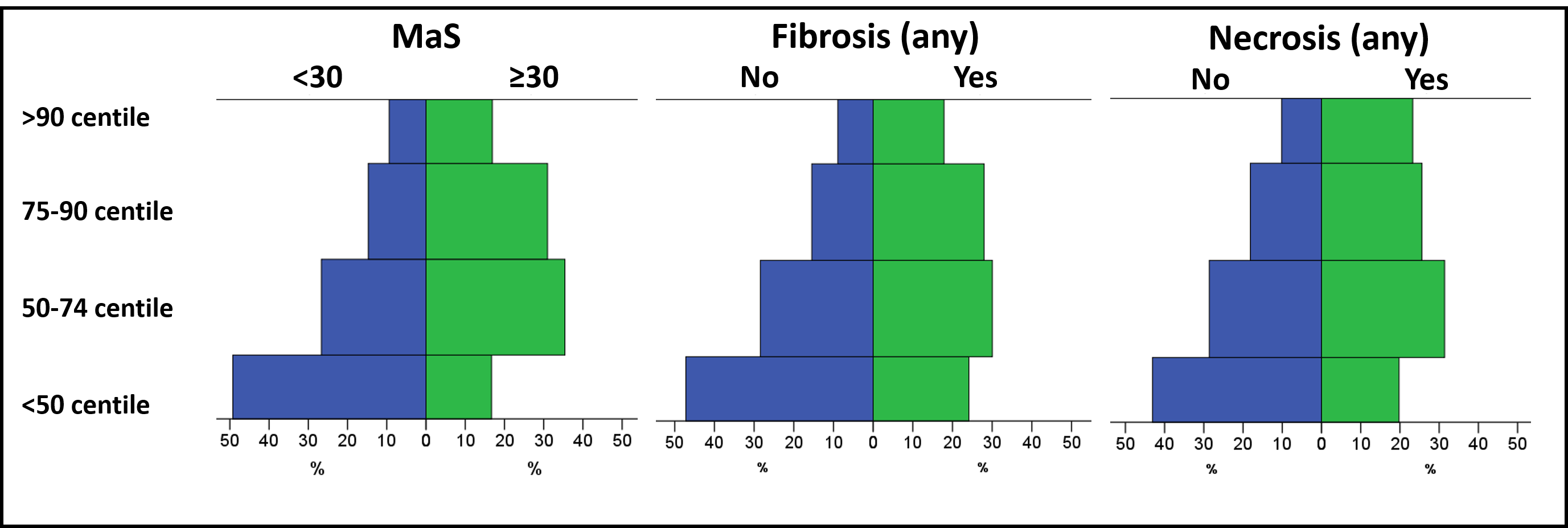

Supplement: Supplementary file 4 — Supplementary file4 (TIF 423 KB) [file 13304_2022_1262_MOESM4_ESM.tif]

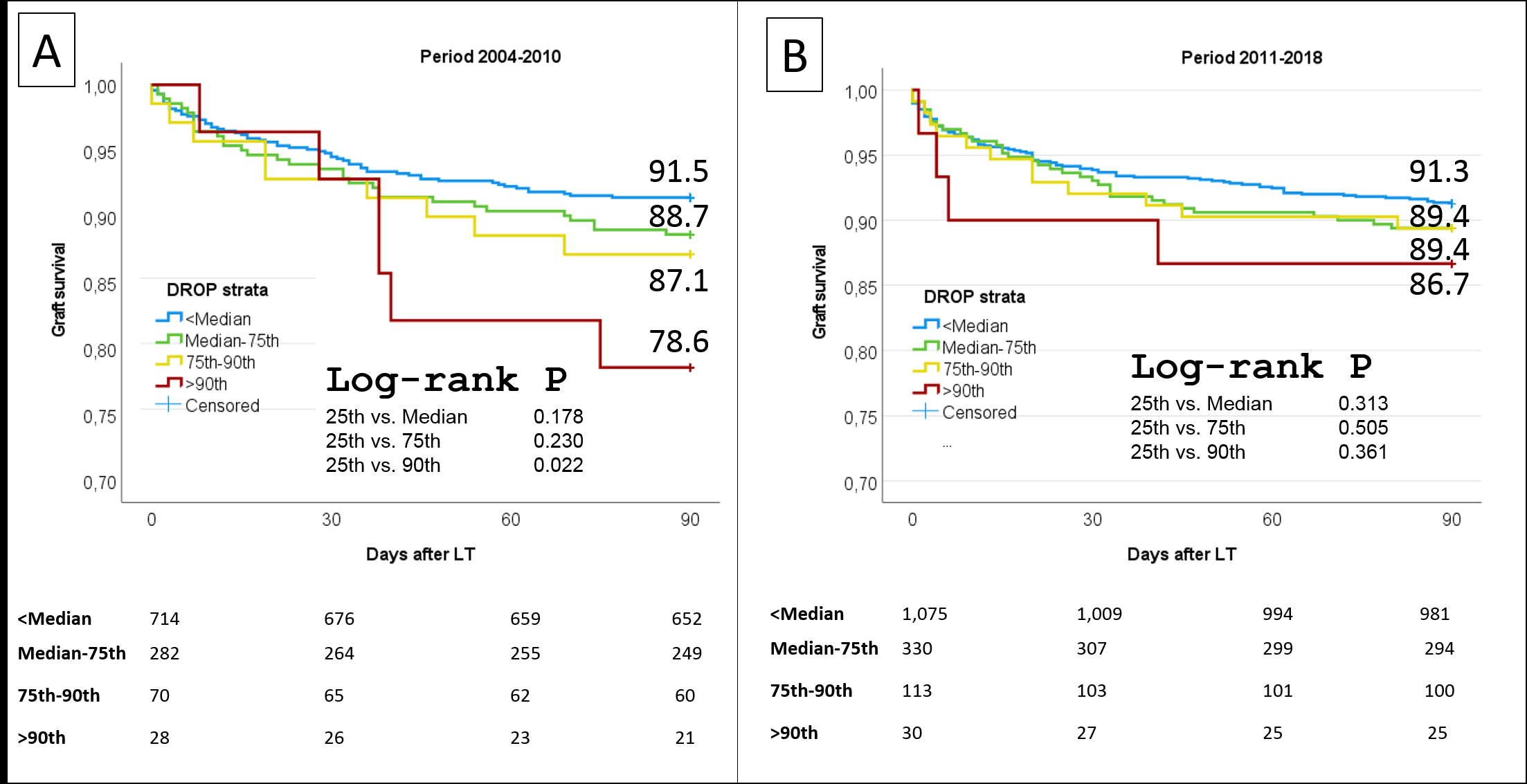

Supplement: Supplementary file 5 — Supplementary file5 (TIF 7323 KB) [file 13304_2022_1262_MOESM5_ESM.tif]
